# Flunixin meglumine tissue residues after intravenous administration in goats

**DOI:** 10.3389/fvets.2023.1341779

**Published:** 2024-01-09

**Authors:** Claire B. Giles, Farha Ferdous, Jennifer L. Halleran, Jim L. Yeatts, Ronald E. Baynes, Danielle A. Mzyk

**Affiliations:** Department of Population Health and Pathobiology, North Carolina State University College of Veterinary Medicine, Raleigh, NC, United States

**Keywords:** flunixin, goat, residues, tolerance limit method, withdrawal interval, withdrawal time

## Abstract

**Background:**

Flunixin is commonly used in goats in an extra-label manner, indicating a significant need to determine withdrawal intervals for edible tissues.

**Objective:**

The objectives of the present study were to investigate the depletion of flunixin meglumine in various goat tissues, including the liver, kidney, fat, and muscle.

**Methods:**

Twenty Boer goats were enrolled and administered an intravenous dose (2.2 mg/kg) of flunixin meglumine. Five animals were randomly euthanized at 24, 48, 72, or 96 h following dosing. All samples were analyzed via ultra-performance liquid chromatography coupled with mass spectrometry.

**Results:**

The concentration of flunixin in all tissues declined rapidly, with the highest mean concentrations quantified in the kidney (0.137 ± 0.062 μg/g) and liver (0.077 ± 0.029 μg/g) tissues at 24 h.

**Conclusion:**

Since any detection of flunixin residues at slaughter found in goat tissues is considered a violative residue, a conservative withdrawal interval of 17 days was calculated to ensure levels of flunixin fell below the regulatory limits of detection in liver, kidney, and muscle tissues.

## 1 Introduction

Flunixin meglumine is a non-steroidal anti-inflammatory drug that is approved by the U.S. Food and Drug Administration (FDA) for the treatment of inflammatory conditions in cattle, horses, and swine. Although transdermal flunixin has been recently approved to control pain associated with foot rot in cattle, there is no approved label for use in any small ruminant species. To effectively treat sheep and goats, veterinarians must often use or prescribe products that are labeled for other species. Any use of flunixin meglumine in goats is considered extra-label drug use. The Animal Medicinal Drug Use Clarification Act of 1994 (AMDUCA) in the United States allows for extra-label use of FDA-approved drugs by or under the supervision of a licensed veterinarian within a valid veterinary-client-patient relationship (1). AMDUCA allows veterinarians to prescribe the use of certain approved animal and human drugs for food-producing animals under specific conditions and limitations (1).

The withdrawal time (WDT) is the period following the last treatment with the drug during which the animal may not be offered for slaughter (2). The length of the withdrawal period is based upon the time necessary for drug residues in the animal to deplete the levels that are shown to fall below the tolerance established by a regulating body (3). In countries outside of the United States, the maximum residue limit (MRL) is often similar to the US tolerance with respect to its calculation and interpretation (4). The WDT is the time point following administration of the labeled dose of a drug after which there is 95% confidence that 99% of treated animals in the reference population will have tissue residues less than the tolerance for that drug, and its calculation is known as the tolerance limit method (TLM) (5). Every approved livestock drug has an approved withdrawal time, which only applies when the drug is used according to the labeled directions. When a medication is used in an extra-label manner to satisfy the conditions of AMDUCA, an extended withdrawal interval (WDI) must be scientifically based. The WDI (in this context) is a scientifically derived recommended withholding period for meat or milk products from animals following the administration of a drug in an extra-label manner. The tolerances for flunixin established for target tissues in cattle (liver and muscle) are based on the assessment of risk to human health and flunixin residue data (3). In small ruminants, flunixin has no established tolerance, meaning that any residue detected is violative.

The US tolerance for flunixin is 0.125 μg/g for liver and 0.025 μg/g for muscle in cattle. The quantification of drug levels in tissues is determined by the U.S. Food Safety and Inspection Service (FSIS). Although there is no tolerance for flunixin in goats, the FSIS has determined the minimum level of applicability (MLA) as the lowest level at which an analytical method has been successfully validated for a residue in each matrix (tissue). It also refers to the lowest level at which a laboratory analyst is expected to maintain ongoing proficiency in the method (6). There is no reported MLA for flunixin in goat liver, but the MLA for goat muscle (0.0125 μg/g) is half of the tolerance allowed from the same tissues in cattle (0.025 μg/g) (7). The MLA for kidneys from goats is the same as muscle (0.0125 μg/g), but there is no tolerance established for kidney tissues from cattle.

Flunixin and related residues have been investigated previously by others in urine (8), plasma (8), serum (9), and milk (10, 11) from treated animals. Currently, there is little data published outlining the relationship between flunixin concentration in the muscles, kidneys, liver, and fat of goats. Therefore, the objective of this study was to determine concentrations of flunixin in various target tissues, including liver, muscle, kidney, and fat tissue, at different times to estimate a withdrawal interval utilizing the FDA’s TLM.

## 2 Materials and methods

### 2.1 Animals and housing

This study was approved by the North Carolina State University Institutional Animal Care and Use Committee (Protocol #20–497; 12 November 2020). Twenty Boer goats (10 wethers and 10 does) between 5 and 8 months of age with weights of 29.2 + 3.1 kg were enrolled in this study. The U.S. FDA recommends a slaughter withdrawal be calculated from at least 20 animals, with at least five animals slaughtered at four separate time points during the expected elimination phase of the drug. All animals were acquired from the North Carolina State University Small Ruminant Education Unit and transferred to the North Carolina State University College of Veterinary Medicine, where they were housed in group housing pens. Goats were fed commercial goat feed (Purina Animal Nutrition, Arden Hills, MN, United States) twice a day and had free access to water and coastal Bermuda grass hay *ad libitum* throughout the study. None of the animals had any previous disease or medical history or administration of non-steroidal anti-inflammatory medications. Physical exams were completed by a veterinarian 24 h prior to dosing to make sure that no marked disease conditions or abnormal clinical signs were present.

### 2.2 Drug administration

Twenty-four hours before the beginning of the study, the goats were restrained for intravenous catheterization, and a 16-gauge intravenous catheter (MILA International, Inc., Florence, KY) was placed into the right jugular vein using the sterile technique. The goats were each weighed on a digital scale the morning of the study to record their weight for determining drug dosage. Injectable flunixin was administered at a single dose of 2.2 mg/kg intravenously (Flunixiject, 50 mg/mL, Henry Schein Animal Health, Dublin, OH).

### 2.3 Tissue collection

Goats were selected for euthanasia at 24, 48, 72, and 96 h based on a randomized study design. Each goat was euthanized via intravenous administration of 87 mg/kg of pentobarbital sodium and phenytoin sodium (Euthasol Euthanasia Solution (390 mg/mL); Virbac Animal Health, Inc. Westlake, TX, United States) after intravenous sedation with 0.5 mg/kg xylazine (Rompun^®^ xylazine injection (100 mg/mL); Dechra Veterinary Products, Overland Park, KS, United States). Samples of the gluteo biceps, subcutaneous fat, entire liver, and both kidneys were taken from each goat postmortem for drug concentration analysis. Before freezing, liver samples were laid out so that two approximately 5 cm diameter punches could be taken out of a cross-section of each lobe from the liver (caudate, quadrate, right, and left lobes) and frozen in Whirl-Pak^®^ (Whirl-Pack Filtration Group, Chicago, IL, United States) bags for ease of processing. The entire remaining liver tissue was also frozen separately to preserve any remaining tissue. All four tissue types were processed and stored at −20°C until analysis. Plasma samples were collected as part of a separate study that is part of a larger population-based model we are developing and were evaluated separately.

### 2.4 Liver, kidney, muscle, and adipose tissue sample preparation

All tissue samples were prepared in the same manner to be analyzed in triplicate. Liver samples were taken from the pre-packaged frozen circular punches, blended together, and weighed into 0.2 g samples. Each 0.2 g sample of goat tissue was placed into a 2 mL bead mill tube and centrifuged at 10,000 × *g* for 30 s to position the tissue in the bottom of the tube. The samples were then spiked with 10 μL of the internal standard, flunixin-d3 (VETRANAL^®^, MilliporeSigma, Burlington, MA, United States), and allowed to sit for 15 min; and 1 mL of 85:15 acetonitrile:ultra-pure water +0.2% formic acid was added to each tube containing the samples (Acetonitrile: Fisher Chemical, Fisher Scientific, ≥99.9% purity; Formic Acid UHPLC Grade: Fisher Chemical, Fisher Scientific, ≥99.9%). The tubes were placed on a FisherBrand™ Bead Mill 24 Homogenizer (Thermo Fisher, Waltham, MA, United States) and programmed to run at 5.00 m/s for 15 s, three times, with a 10 s rest between cycles. The tubes were taken out of the homogenizer and placed in a microcentrifuge at 10,000 × *g* for 7 min. After centrifugation, 800 μL of the supernatant was loaded onto a Waters Oasis^®^ 1cc (30mg) MCX cartridge (Waters Corporation, Milford, MA, United States) for solid-phase extraction (SPE). Each sample was washed with 1 mL of 0.2% formic acid in water, followed by a second wash with 1 mL of 100% methanol (Optima LC/MS, Fisher Chemical, Fisher Scientific, >99.9% purity). New, clean 16 × 100 mm borosilicate glass tubes were placed under each sample before eluting with 1 mL of 5% ammonium hydroxide (Certified ACS Plus, Fisher Chemical, Fisher Scientific, ≥28–30%; 14.8 N, pH 12) in methanol. Each sample was evaporated to dryness using a nitrogen evaporator (RapidVap Vertex Evaporater, Labconco, Kansas City, MO, United States) at 55°C for approximately 10 min, reconstituted in 300 μL of 1:1 acetonitrile:ultra-pure water, vortexed for 30 s. Finally, all samples were filtered through devices containing 0.2 μm PVDF filter media (Whatman Mini-UniPrep™ syringeless filters, Cytiva, Marlborough, MA, United States) before analysis via ultra-performance liquid chromatography coupled to mass spectrometry (UPLC/MS).

### 2.5 Validation and UPLC/MS conditions

Method validation was performed according to the FDA Bioanalytical Guidelines (12). Standard curves for each tissue analysis were prepared by fortifying untreated tissue homogenate with flunixin standard (Sigma-Aldrich, MilliporeSigma, 96.9% purity), which produced a linear concentration range of 1–500 ng/g with a correlation coefficient, *R*^2^ of 0.99. Recovery, accuracy, and precision were determined by analyzing five replicates at low, medium, and high concentrations within the concentration range of the curve for each tissue. Intraday precision and accuracy were obtained by analyzing three different flunixin concentrations repeated five times each on the same day. Interday precision and accuracy were obtained by analyzing seven different concentrations on five different days. Intra- and interday precision and accuracy are shown in the Supplementary materials. The limit of detection (LOD) for liver, muscle, and fat samples was determined to be 0.001 μg/g, and the limit of quantification (LOQ) was determined to be 0.002 μg/g for these tissues. The LOD for kidney samples was determined to be 0.002 μg/g, and the LOQ was determined to be 0.005 μg/g.

The analysis was performed on a Waters Ultra-Performance Liquid Chromatograph coupled to a Waters Acquity Qda mass spectrometer detector (Waters Corporation, Milford, MA, United States). The instrument was set to single ion recording of 297 m/z and 300 m/z for flunixin and flunixin-d3, respectively, using electrospray ionization in the positive ion mode (ESI+). The cone voltage was 20V. A Waters Acquity UPLC BEH C18 1.7 μm (2.1 mm × 50 mm) column with corresponding VanGuard™ Pre-Column (2.1mm × 5mm column, Waters Corporation, Milford, MA, United States) was used for all separations involving the liver. For all other tissues, a Waters Acquity UPLC BEH Phenyl 1.7 μm (2.1 mm × 100 mm) column with corresponding VanGuard™ Pre-column (2.1mm × 5 mm) was used. The mobile phase was a gradient. Solvent A1 was 0.1% formic acid in water. Solvent A2 was 90:10 ultra-pure water:acetonitrile. Solvent B1 was 0.1% formic acid in acetonitrile. The flow rate was 0.40 mL/min. For all tissues except the kidney, the gradient was programmed as follows: From 0.00 to 1.00 min, the composition was 70% A1:30% B1; from 1.00 to 2.50 min, the composition changed linearly to 10% A1:90% B1, then held until 3.50 min; finally, back to 70% A1:30% B1 at 3.51 min and held to 5.00 min. For the kidney, the gradient was programmed as follows: From 0.00 to 0.50 min, the composition was 70% A2:30% B1; from 0.50 to 4.00 min, the composition changed linearly to 10% A2:90% B1, then held until 4.50 min; finally, back to 70% A2:30% B1 at 4.51 min and held to 6.00 min.

### 2.6 Tissue elimination half-life calculations

The tissue elimination half-life refers to an estimate of the time at which the concentrations or the amount of the drug in that particular tissue will be reduced by exactly one-half in the terminal phase of a concentration–time curve. Although the half-life indicates a 50% reduction of the concentration of the drug when pseudo equilibrium has been achieved, it does not provide any assurance that the drug is being eliminated from the body; therefore, estimating a WDI based on the half-life will be misleading. However, the half-life provides an understanding of how long the drug will remain in the tissue or how often the drug should be administered to have the desired level of efficacy. Therefore, to estimate elimination half-life, the most commonly used formula is t½ = 0.693/k, where k is the slope obtained by fitting a simple linear regression model of log of concentrations vs. time from tissue concentrations data set.

### 2.7 Withdrawal interval calculations

The TLM calculation uses the ordinary least squares method to fit the simple linear regression of the log of concentrations vs. time profiles from tissue concentrations (5). The assumptions, namely the correct specification of the mean model, the homoscedastic/equal variance assumption, uncorrelated errors, and the normality assumption, must be satisfied to fit the linear regression model. Furthermore, based on the fitted values, the 99% upper tolerance limit with 95% confidence can be calculated at any time point. The values of the concentration predicted at the new time point are compared to the target tolerance. The time with a corresponding concentration less than or equal to the specified tolerance is then reported as the withdrawal time. Although the calculations to determine the WDI for this study were based on the federal regulatory method used in the United States, administering flunixin to goats is considered extra-label and therefore has no federally approved withdrawal time. To determine a WDI using the TLM, a limit of detection (assay sensitivity) was assigned a tolerance level and entered into the calculation. To determine a recommended WDI on the examined edible tissues, several different scenarios were evaluated using the LODs from either our lab (FARAD, Food Animal Residue Avoidance Databank), FSIS MLA, or the tolerance in tissue from cattle by the FDA. Specific evaluations of each scenario can be seen in the Supplementary materials. The final parameters for the WDI model used the FARAD assay sensitivity as the LOD and tolerance for each tissue (Table 1).

**Table 1:** FARAD limit of detection/tolerance levels for withdrawal interval calculations.

Tissue	Limit of detection (μg/g)	Tolerance^a^ (μg/g)
Liver	0.001	0.001
Kidney	0.002	0.002
Muscle	0.001	0.001
Fat	0.001	0.001

a Used for parameter inclusion to determine the WDI in the selected model and does not indicate an approved tolerance in any goat tissues.

## 3 Results

### 3.1 Tissue concentrations and elimination-half lives

**Figure 1:**
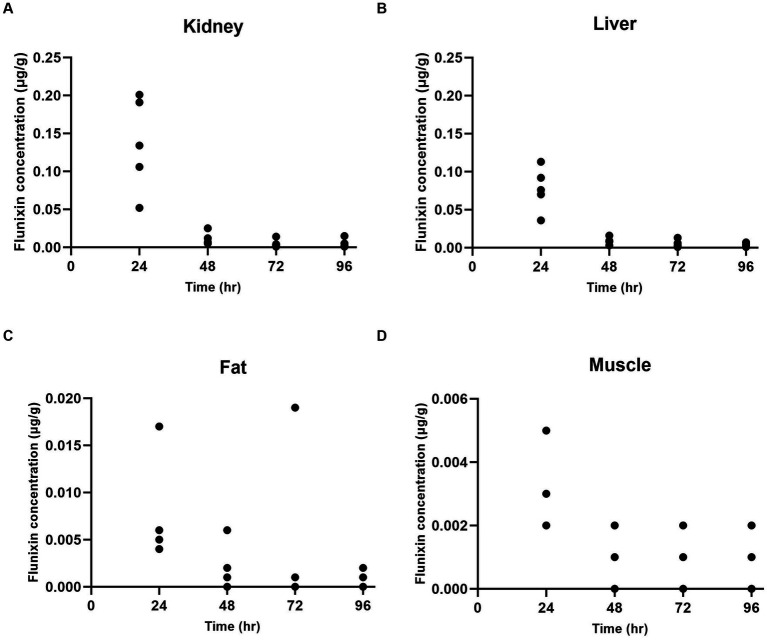
Individual flunixin concentrations in goats (*n* = 5) at each time point in the kidney **(A)**, liver **(B)**, fat **(C)**, and muscle **(D)** tissues.

At 24 h after administration, the highest concentration of flunixin was found in the kidney (Figure 1A) and liver (Figure 1B) tissues, followed by fat (Figure 1C) and muscle (Figure 1D) tissues. Flunixin tissue concentration levels declined rapidly from the tissues, with the highest mean concentrations seen in the kidney (0.137 ± 0.062 μg/g) and liver tissues (0.077 ± 0.029 μg/g) at 24 h after IV administration. Flunixin was not detected from any tissue above the bovine liver/muscle tolerance or FSIS MLA at 96 h. The tissue elimination half-lives for flunixin in goats were highest in the muscle (55.81 h), followed by fat (36.15 h), liver (14.34 h), and kidney (13.65 h).

### 3.2 Withdrawal interval

**Figure 2:**
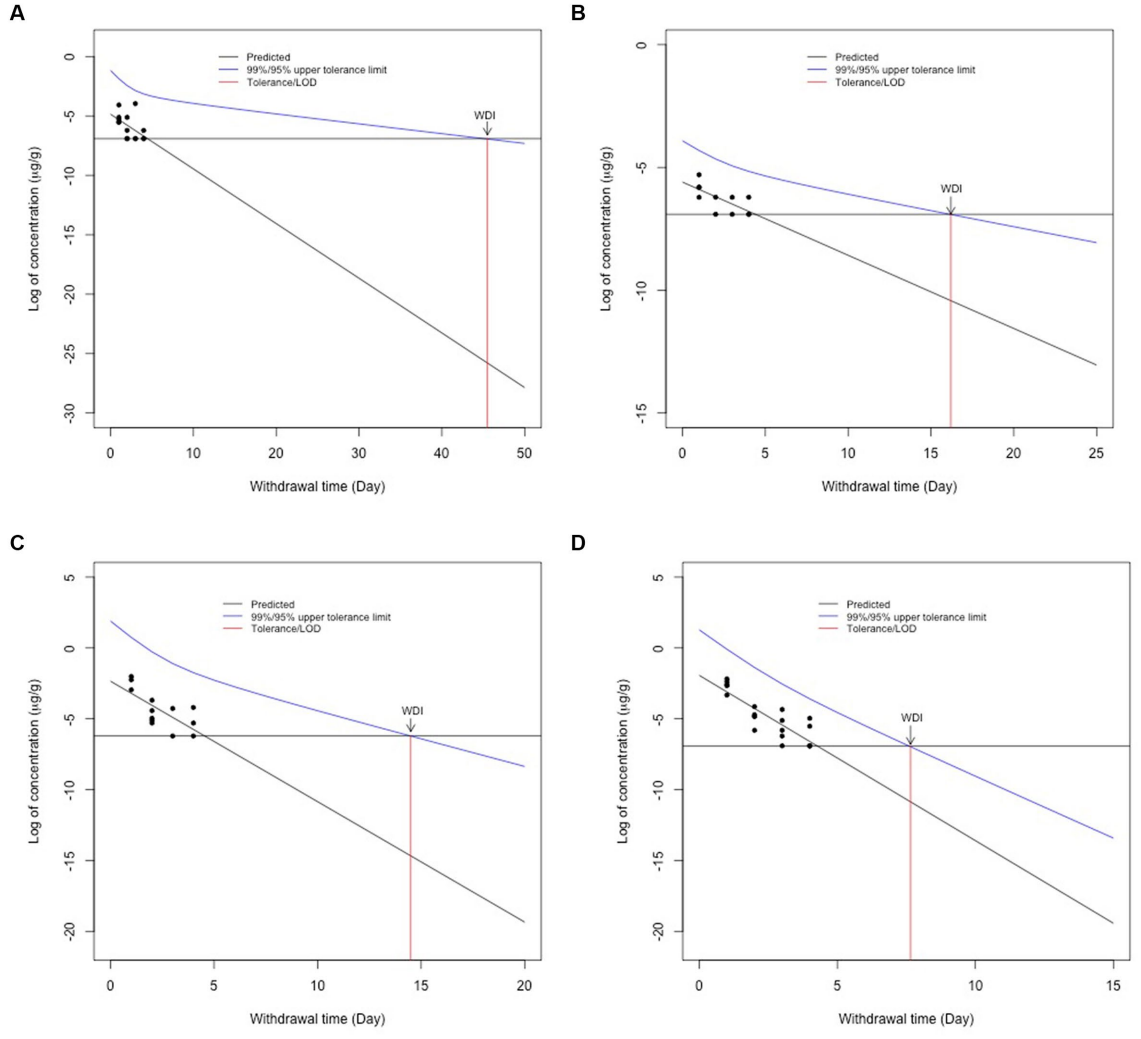
Withdrawal interval calculations for fat **(A)**, muscle **(B)**, kidney **(C)**, and liver **(D)** following the FDA tolerance limit method following 2.2 mg/kg intravenous flunixin. Black dots represent flunixin concentrations from individual animals at each time point. The blue line represents the 95% confidence interval for when 99% of the population has a drug concentration below a given target concentration. The red line represents the WDI for each tissue when the concentration falls below the FARAD limit of detection for each tissue.

Using the LOD validated for each goat tissue in our lab (Table 1), the tolerance for allowable concentrations in goat tissues was set at 0.001 μg/g (liver, muscle, and fat) and 0.002 μg/g for kidney. For WDI calculations, all data points that were above the FARAD lab LOD for each tissue were included in the calculations. Using these values, a semi-linear regression was performed, and the longest WDI was determined in fat tissue (46 days; Figure 2A), followed by muscle (17 days; Figure 2B), kidney (15 days; Figure 2C), and liver (8 days; Figure 2D). All values for WDI were rounded up to the nearest whole number.

## 4 Discussion

This study was performed to establish a WDI in meat goats following the administration of a single intravenous dose of 2.2 mg/kg of flunixin meglumine utilizing the FDA TLM. Flunixin concentrations found in the liver and muscle of all goats were quantified below the established FDA tolerance limit for flunixin in cattle and in the liver (0.125 μg/g) and muscle (0.025 μg/g) of adult cows at all time points. The mean flunixin concentrations in kidneys were the highest levels of any tested tissues at 24 h after administration (0.137 μg/g). Although fat tissue does not have an MLA set by the FSIS, we evaluated the concentrations based on the confirmatory testing level for kidney and muscle. Flunixin was detected in two fat samples above 0.0125 μg/g at 24 and 72 h. This is likely due to the variability of drug distribution and tissue binding and elimination in each goat.

In accordance with the FDA’s Guidance for Industry, the TLM considers the rate of depletion and variability among individual animals to determine the 95% confidence interval for when 99% of the population will have a drug concentration below a given target concentration (5). The limit of detection for FARAD’s assay was much lower than the sensitivity reported by the FSIS MLA. The MLA for fat is not available from FSIS (personal communication with askFSIS) and, therefore, no WDI could be determined using assay sensitivity from FSIS testing methods. Since flunixin given intravenously at the labeled cattle dose is considered an extra-label use, it is imperative to acknowledge that any detection of flunixin residue at the time of slaughter in the goat tissues is considered a violative residue, so a more conservative withdrawal interval is needed. Elimination half-lives determined in cattle were longer in the liver (34.2 h) and kidney (29.6 h) compared to values determined in the goat tissues. The reported differences between the FARAD assay sensitivity or limit of the detection and the FSIS MLA for each tissue may contribute to differences noted between withdrawal interval calculations. The elimination half-life has been described to be the most susceptible to the sensitivity of the analytical method (13). The muscle tissue elimination half-life determined in goats from this study (55.81) is approximately two times longer than previously noted in cattle (14). The analytical method with a lower detection limit has been shown to affect the terminal part of the tissue depletion curve because the concentrations can be detected more accurately for longer periods. The increased sensitivity of the method in our study, in addition to the lower tolerance selected (based on LOD) as compared with the FSIS MLA, likely contributed to a longer elimination half-life in muscle tissue and reported withdrawal interval.

Administration of multiple doses of flunixin by a route other than IV may require extending the WDI for target tissues to ensure that violative residues are not detected in treated goats. It should also be noted that these goats were healthy, and the metabolism of flunixin could differ greatly in a diseased animal, and our estimates provided here may not be applicable to unhealthy goats (14, 15).

The FDA tolerance limit method uses the linear regression model based on which the 99% tolerance with 95% confidence is calculated using the non-central t-distribution. Therefore, to fit the linear regression model, the data set involving the log of concentrations and time needs to satisfy the assumptions, namely the correct specification of the mean model, homoscedasticity, uncorrelated errors, and normality. The violation of these assumptions leads to the removal of some data points so that the assumptions can be satisfied, which leads to the exclusion of important information from the entire data. If we excluded the concentrations that were lower than the FSIS detection limits for goat tissues, only 15 out of 100 samples (including all tissues from all 20 goats) would have been included in the analysis. It is important to note that removing points below the tolerance, assay limit of detection, and/or MLA can decrease the withdrawal time estimations, as noted with the WDI calculated using FSIS MLAs. By removing the time points that most diminish linearity, the WDI can also be decreased. Another potential issue is that by removing data points, the WDI could also be increased due to a change in the degree of freedom of the data, which increases the size of the confidence interval surrounding the calculated population’s t-distribution. In addition, limits of detection change as analytical methods improve and may impact future withdrawal interval calculations.

This study provides new tissue depletion data following the use of a single dose of flunixin intravenously in Boer goats. These data have been used to create a slaughter WDI recommendation of at least 17 days for muscle, liver, and kidney tissue, which is critical for protecting the food supply following extra-label drug use in goats. Despite no current regulatory testing method to detect flunixin in adipose tissue, a longer withdrawal interval (46 days) is recommended for fat due to the variability in residues found in goats. This study reports major differences in WDI calculations based on assay sensitivities in different tissues. Further studies should investigate the concentrations of flunixin in diseased animals that were administered flunixin to determine if tissue concentrations and elimination rates differ in healthy vs. clinically diseased goats.

## Data availability statement

The raw data supporting the conclusions of this article will be made available by the authors, without undue reservation.

## Ethics statement

The animal study was approved by North Carolina State University Institutional Animal Care and Use Committee. The study was conducted in accordance with the local legislation and institutional requirements.

## Author contributions

CG: Conceptualization, Data curation, Formal analysis, Investigation, Methodology, Validation, Visualization, Writing – original draft, Writing – review & editing. FF: Data curation, Formal analysis, Visualization, Writing – original draft, Writing – review & editing. JH: Data curation, Formal analysis, Investigation, Methodology, Supervision, Writing – review & editing. JY: Formal analysis, Validation, Writing – review & editing. RB: Conceptualization, Formal analysis, Funding acquisition, Investigation, Methodology, Project administration, Resources, Supervision, Validation, Visualization, Writing – review & editing. DM: Conceptualization, Data curation, Formal analysis, Funding acquisition, Investigation, Methodology, Project administration, Supervision, Validation, Visualization, Writing – original draft, Writing – review & editing.
