# Supplementary material for: Flunixin meglumine tissue residues after intravenous administration in goats
Source: Front Vet Sci. 2024 Jan 9;10:1341779. doi: 10.3389/fvets.2023.1341779 (PMC10803588; doi:10.3389/fvets.2023.1341779)
Supplement: Supplementary file 1 [file Data_Sheet_1.docx]

Supplementary Material

**Flunixin meglumine tissue residues after intravenous administration in goats**

Claire B Giles, Farha Ferdous, Jennifer L. Halleran, Jim L. Yeatts, Ronald E. Baynes, Danielle A. Mzyk^*^

*** Correspondence:** Danielle A. Mzyk, dalindqu@ncsu.edu

# Supplementary Data - *Intraday precision and accuracy*

## Intraday precision and accuracy

### Liver

| Spiked Flunixin  Concentration in Liver  (µg/g) | Average Concentration in Liver (µg/g)  n=5 | Standard Deviation  (µg/g) | Relative Standard Deviation  (RSD%) | Average Accuracy (%) |
| --- | --- | --- | --- | --- |
| 0.002 | 0.0020 | 0.0002 | 10.2 | 99.5 |
| 0.005 | 0.0042 | 0.0001 | 3.1 | 84.7 |
| 0.010 | 0.0088 | 0.0003 | 3.3 | 88.5 |

### Kidney

| Spiked Flunixin  Concentration in Kidney  (µg/g) | Average Concentration in Kidney (µg/g)  n=5 | Standard Deviation  (µg/g) | Relative Standard Deviation  (RSD%) | Average Accuracy (%) |
| --- | --- | --- | --- | --- |
| 0.002 | 0.0017 | 0.0001 | 3.5 | 86.9 |
| 0.005 | 0.0046 | 0.0002 | 3.8 | 91.2 |
| 0.010 | 0.0096 | 0.0005 | 5.1 | 95.5 |

### Muscle

| Spiked Flunixin  Concentration in Muscle  (µg/g) | Average Concentration in Muscle (µg/g)  n=5 | Standard Deviation  (µg/g) | Relative Standard Deviation  (RSD%) | Average Accuracy (%) |
| --- | --- | --- | --- | --- |
| 0.002 | 0.0020 | 0.0001 | 6.4 | 100.0 |
| 0.005 | 0.0051 | 0.0002 | 3.2 | 101.4 |
| 0.010 | 0.0103 | 0.0004 | 4.3 | 103.1 |

### Fat

| Spiked Flunixin  Concentration in Fat  (µg/g) | Average Concentration in Fat (µg/g)  n=5 | Standard Deviation  (µg/g) | Relative Standard Deviation  (RSD%) | Average Accuracy (%) |
| --- | --- | --- | --- | --- |
| 0.002 | 0.0020 | 0.0001 | 5.7 | 98.1 |
| 0.005 | 0.0047 | 0.0003 | 5.8 | 94.1 |
| 0.010 | 0.0093 | 0.0003 | 2.8 | 93.5 |

## Interday precision and accuracy

### Liver

| Spiked Flunixin  Concentration in Liver  (µg/g) | Average Concentration in Liver (µg/g)  n=5 | Standard Deviation  (µg/g) | Relative Standard Deviation  (RSD%) | Average  Accuracy (%) |
| --- | --- | --- | --- | --- |
| 0.001 | 0.001 | 0.0001 | 9.1 | 104.4 |
| 0.002 | 0.002 | 0.0002 | 8.9 | 101.5 |
| 0.005 | 0.005 | 0.0003 | 6.6 | 96.5 |
| 0.01 | 0.010 | 0.0004 | 4.1 | 99.4 |
| 0.05 | 0.048 | 0.0036 | 7.5 | 96.8 |
| 0.1 | 0.101 | 0.0055 | 5.5 | 101.3 |
| 0.5 | 0.500 | 0.0075 | 1.5 | 100.1 |

### Kidney

| Spiked Flunixin  Concentration in Kidney  (µg/g) | Average Concentration in Kidney (µg/g)  n=5 | Standard Deviation  (µg/g) | Relative Standard Deviation  (RSD%) | Average  Accuracy (%) |
| --- | --- | --- | --- | --- |
| 0.001 | 0.002 | 0.0007 | 43.4 | 155.7 |
| 0.002 | 0.002 | 0.0003 | 14.2 | 109.2 |
| 0.005 | 0.005 | 0.0001 | 2.5 | 94.7 |
| 0.01 | 0.009 | 0.0006 | 6.5 | 90.4 |
| 0.05 | 0.049 | 0.0030 | 6.0 | 98.6 |
| 0.1 | 0.101 | 0.0051 | 5.1 | 100.6 |
| 0.5 | 0.515 | 0.0311 | 6.0 | 103.0 |

### Muscle

| Spiked Flunixin  Concentration in Muscle  (µg/g) | Average Concentration in Muscle (µg/g)  n=5 | Standard Deviation  (µg/g) | Relative Standard Deviation  (RSD%) | Average  Accuracy (%) |
| --- | --- | --- | --- | --- |
| 0.001 | 0.001 | 0.0001 | 7.1 | 111.8 |
| 0.002 | 0.002 | 0.0001 | 4.3 | 98.2 |
| 0.005 | 0.005 | 0.0003 | 5.5 | 97.7 |
| 0.01 | 0.009 | 0.0004 | 4.4 | 94.6 |
| 0.05 | 0.049 | 0.0019 | 3.9 | 98.0 |
| 0.1 | 0.099 | 0.0032 | 3.3 | 99.3 |
| 0.5 | 0.502 | 0.0041 | 0.8 | 100.5 |

### Fat

| Spiked Flunixin  Concentration in Fat  (µg/g) | Average Concentration in Fat (µg/g)  n=5 | Standard Deviation  (µg/g) | Relative Standard Deviation  (RSD%) | Average  Accuracy (%) |
| --- | --- | --- | --- | --- |
| 0.001 | 0.001 | 0.00003 | 2.3 | 109.8 |
| 0.002 | 0.002 | 0.00007 | 3.5 | 101.5 |
| 0.005 | 0.005 | 0.00025 | 5.3 | 94.2 |
| 0.01 | 0.010 | 0.00039 | 4.0 | 96.5 |
| 0.05 | 0.049 | 0.00136 | 2.8 | 97.0 |
| 0.1 | 0.101 | 0.00341 | 3.4 | 100.6 |
| 0.5 | 0.501 | 0.00354 | 0.7 | 100.3 |

# Supplementary Data – Withdrawal Interval Calculations

The authors also explored estimating the withdrawal interval in different scenarios using the FDA tolerance limit method. Across all the scenarios, we have considered all the concentrations that are above/at the LOD, however we explored several different tolerances selected as the permitted concentration such as FARAD limit of detection/limit of quantification, US tolerance. In addition to this, as the USDA Food Safety and Inspection Service (FSIS) considers any residue of flunixin in goats at the slaughter time as the violative residues and in this study we are using flunixin in an extra label manner in goats, therefore it is important to estimate the withdrawal intervals by utilizing the FSIS LOD or FSIS LOQ as the safe/permitted concentration. The following is a brief discussion of estimating the WDI’s in each of the scenarios.

## **Scenario 1**

To estimate the WDI, the FARAD limit of detection/ tolerance levels for liver 0.001 (µg/g), kidney 0.002 (µg/g), muscle 0.001 (µg/g), and fat 0.001 (µg/g) were used as the tolerance in the respective tissues. The following table provides the WDI estimated in this scenario.

| Scenario 1 | |
| --- | --- |
| Tissues | WDI (days) |
| Liver | 8 |
| Kidney | 15 |
| Muscle | 17 |
| Fat | 46 |

## Scenario 2

| Scenario 2 | |
| --- | --- |
| Tissues | WDI (days) |
| Liver | 7 |
| Kidney | 13 |
| Muscle | 11 |
| Fat | 37 |

To estimate the WDI, the FARAD limit of quantification (LOQ) for liver 0.002 (µg/g), kidney 0.005 (µg/g), muscle 0.002 (µg/g), and fat 0.002 (µg/g) were used as the tolerance/permitted concentration in the respective tissues. The following table provides the WDI estimated in this scenario.

## Scenario 3

## To estimate the WDI, as there are no established US tolerances for flunixin in goats, therefore the US tolerance for flunixin in cattle which is 0.125 µg/g for liver and 0.025 µg/g for muscle were used as the tolerance/permitted concentration. In addition, as there is no US tolerance established for kidney and fat in cattle, hence we have used the same liver tolerance 0.125 µg/g for kidney and the same muscle tolerance 0.025 µg/g for fat as the permitted concentration. The following table provides the WDI estimated in this scenario.

| Scenario 3 | |
| --- | --- |
| Tissues | WDI (days) |
| Liver | 3 |
| Kidney | 5 |
| Muscle | 0 |
| Fat | 8 |

## Scenario 4

To estimate the WDI, the FSIS limit of detection/MLA was used as the permitted concentration. The FSIS LOD of flunixin in cattle liver is 0.0313 µg/g, therefore, to calculate the WDI in liver and kidney in goat we used 0.0313 µg/g as the permitted concentration. As there is no established LOD for muscle/fat by FSIS, therefore we did not estimate any withdrawal intervals in the respective tissues in this scenario. The following table provides the WDI estimated in this scenario.

| Scenario 4 | |
| --- | --- |
| Tissues | WDI (days) |
| Liver | 4 |
| Kidney | 8 |
| Muscle | - |
| Fat | - |

## Scenario 5

| Scenario 5 | |
| --- | --- |
| Tissues | WDI (days) |
| Liver | 4 |
| Kidney | 7 |
| Muscle | 2 |
| Fat | 15 |

To estimate the WDI, the FSIS limit of quantification/LOQ was used as the permitted concentration. The FSIS LOQ for flunixin in bovine liver and muscle are 0.0625 µg/g and 0.0125 µg/g, respectively. As there are no established tolerances for flunixin in goats, therefore we have used the bovine liver LOQ as the permitted concentrations for both the liver and kidney and bovine muscle LOQ as the permitted concentration for both the muscle and fat in order to estimate the WDI in goats. The following table provides the WDI estimated in this scenario.
